# F_1_ hybrids in an allopolyploid crop: genomic architecture, introgression mosaics, and breeding innovation—insights from *Coffea arabica*

**DOI:** 10.3389/fpls.2026.1827609

**Published:** 2026-06-19

**Authors:** Benoit Bertrand, Delphine Mieulet, Jean-Christophe Breitler

**Affiliations:** 1 Association for Science and Information on Coffee (ASIC), Scientific Secretary, Paris, France; 2 CIRAD, UMR AGAP Institut, Montpellier, France; 3 UMR AGAP Institut, Univ Montpellier, CIRAD, INRAE, Institut Agro, Montpellier, France; 4 CIRAD, UMR DIADE, Montpellier, France; 5 DIADE, University of Montpellier, IRD, CIRAD, Montpellier, France

**Keywords:** allopolyploidy, Arabica coffee, climate resilience, genomic prediction, heterosis, hybrid breeding, introgression, regulatory heterosis

## Abstract

While hybrid breeding has revolutionized the improvement of many crop species, its mechanistic underpinnings remain poorly understood in genetically constrained allopolyploids. *Coffea arabica*—a predominantly self-pollinating allotetraploid derived from *C. canephora* and *C. eugenioides*—exemplifies this paradox: despite having one of the narrowest genetic bases of any major crop, structured crosses consistently yield strong and predictable heterosis. This raises a critical question: How does hybrid vigor emerge from an allopolyploid genome shaped by long-term pure-line selection? In this review, we synthesize four decades of breeding experiments, quantitative genetics, and recent genomic advances to propose a unified framework of “hybrid activation” in Arabica. We identify two complementary mechanisms driving heterosis: 1. Introgressed-block heterosis, arising from the recombination of divergent chromosomal mosaics introgressed from *C. canephora*, which generates structural and functional complementarity within otherwise uniform genetic backgrounds. 2. Regulatory heterosis stemming from the rebalancing of ancestral subgenome interactions. This leads to non-additive gene expression, enhanced physiological integration and improved environmental buffering, even with limited nucleotide diversity. We explore how these mechanisms interact through dosage effects, homeolog regulation, and genome architecture. Additionally, we highlight how chromosome-scale assemblies, GWAS, haplotype-resolved analyses, and genomic prediction models incorporating non-additive effects can rationally structure heterotic groups and accelerate hybrid design. Meanwhile, clonal propagation systems and emerging male-sterility-based seed technologies are gradually overcoming historical barriers to hybrid deployment in perennial selfing crops, though technical and operational challenges remain. This framework extends beyond coffee, offering a broader perspective on how hybridization can unlock the latent evolutionary potential embedded in allopolyploid genome architecture, thereby contributing to the development of climate-resilient crops.

## Highlights

- Arabica a selfing allopolyploid crops can express strong and predictable heterosis despite severe allelic bottlenecks.
- In *Coffea arabica*, hybrid vigor arises from two complementary mechanisms: introgressed genomic mosaics and subgenome regulatory rebalancing.
- Genome-enabled tools (reference genome resources, GWAS, genomic prediction, and emerging pangenome initiatives) enable the rational identification and structuring of heterotic groups.
- Male-sterility systems and clonal propagation overcome long-standing barriers to hybrid deployment in predominantly selfing crops.
- Hybrid breeding may reactivate hidden evolutionary potential in genetically constrained allopolyploid species.

## 1 Introduction

Hybrid breeding has reshaped modern agriculture, yet its theoretical foundations remain unevenly reconciled across plant systems. In diploid, predominantly outcrossing crops such as maize, heterosis has been successfully exploited through the structuring of heterotic groups and reciprocal recurrent selection (Xiao et al., 2021). By contrast, self-fertilizing crops with narrow genetic bases are generally considered less suitable for hybrid breeding, because classical dominance and overdominance models emphasize segregating allelic variation as the main substrate of hybrid vigor (Paril et al., 2024; Revell et al., 2025).

This expectation becomes particularly intriguing in allopolyploid crops. Formed through interspecific hybridization followed by genome doubling, allopolyploids combine divergent subgenomes within a shared regulatory landscape. Such genome architectures generate redundancy, dosage interactions, and complex transcriptional networks shaped by subgenome dominance and non-additive gene regulation (Yoo et al., 2014). These features may create phenotypic plasticity and regulatory flexibility even when nucleotide diversity is limited.

*Coffea arabica* represents a particularly stringent case. This allotetraploid species originated from hybridization between *C. canephora* and *C. eugenioides* (Lashermes et al., 1999) and subsequently underwent a severe domestication bottleneck, resulting in one of the narrowest genetic bases among major crops (Scalabrin et al., 2020). For more than a century, breeding relied primarily on pure-line selection (Van der Vossen et al., 2015), reinforcing the view that hybrid breeding would offer limited advantages (Krug and Filho, 1950). Yet a growing body of experimental evidence has repeatedly documented strong and often predictable heterosis for yield, vigor, and stress tolerance in well-designed crosses (Medeiros et al., 2021; Andrade et al., 2025). Similar results were obtained in our recent submitted study (Bertrand et al., 2026).

Recent advances in genomics and quantitative genetics have renewed interest in these observations. Genome assemblies, GWAS, and genomic prediction are beginning to clarify how introgressed chromosomal segments and regulatory interactions between subgenomes may contribute to hybrid vigor (Scalabrin et al., 2024). At the same time, innovations in clonal propagation and hybrid seed production are progressively removing practical barriers that historically limited hybrid deployment in coffee (Etienne et al., 2018; Georget et al., 2019).

In this review, we examine how strong and predictable heterosis can emerge in a predominantly selfing allopolyploid species with a narrow genetic base. We first revisit the historical emergence and quantitative validation of heterosis in *Coffea arabica*, tracing the progression from early crossing experiments to modern multi-environment hybrid trials (Section 1). We then explore how functional diversity can arise despite limited allelic variation (Section 2), describe the existence of complementary heterotic systems in Arabica (Section 3), and discuss how genomic tools can structure heterotic groups (Section 4). Finally, we consider the transition from experimental hybrids to scalable hybrid agriculture, propose a broader framework for hybrid activation, and highlight key research gaps and future directions (Sections 5–7).

## 2 Historical emergence and quantitative validation of heterosis in *Coffea arabica*

In 1950, Krug and Filho concluded, after extensive experimentation, that no heterosis for yield was detectable in the Arabica material they investigated. Their analyses were conducted within the extremely narrow Typica–Bourbon genetic base, which limited allelic and structural diversity and therefore constrained the possibility of observing heterotic effects. This early conclusion profoundly shaped subsequent breeding strategies.

Consequently, most national breeding programs adopted pedigree selection as their primary improvement strategy. This approach favored the development of homogeneous pure-line cultivars and reduced seed production costs, reinforcing the perception that Arabica was poorly suited to hybrid breeding.

The first indications that this paradigm might be incomplete emerged from crosses involving genetically differentiated materials. When Ethiopian accessions were crossed with Caturra derivatives, A. Charrier (1978) reported pronounced vigor effects resembling heterosis. Around the same period, breeding programs in East Africa through diallel device (Walyaro, 1983) and in Cameroon (Cilas et al., 1998) demonstrated that hybrid combinations could improve resistance to coffee leaf rust and coffee berry disease (*Colletotrichum kahawae*), providing further empirical evidence that hybrid vigor was biologically meaningful in Arabica.

Subsequent studies confirmed heterotic responses in increasingly complex crossing schemes, including three-way hybrids in Tanzania (Nyange et al., 1999), multi-parental hybrids such as Ruiru 11 in Kenya, and structured hybrid combinations evaluated in Ethiopia through a diallel (Bellachew et al., 1993). Together, these findings progressively challenged the long-standing assumption that heterosis was absent in Arabica.

A major turning point occurred in 1990 with the launch of an F_1_ hybrid breeding program in Central America through a collaboration between CIRAD, PROMECAFE, and CATIE. Results obtained from factorial crosses between introgressed pure lines and Ethiopian genotypes demonstrated that F_1_ hybrids could substantially outperform conventional introgressed pure-line cultivars (Bertrand, 2002; Bertrand et al., 2005). Similar outcomes were later reported in Colombia (Bertrand et al., 2021, 2026 under review). Subsequent multi-location trials conducted by PROMECAFE in Central America or by CIRAD and ECOM in Nicaragua confirmed the superior yield performance and stability of selected hybrids (Bertrand et al., 2006, 2011; Marie et al., 2020). Studies by Pappo et al. (2021) and Kahsay et al. (2023) showed that F_1_ hybrids such as ‘Centroamérica’ can enhance agroecosystem resilience, further highlighting the agronomic relevance of hybrid breeding.

Since around 2015, World Coffee Research (WCR), which represents more than 200 coffee companies worldwide, has actively promoted Arabica F_1_ hybrids (Berny Mier y Teran et al., 2025). The organization has developed new hybrids currently under evaluation and produced educational resources to facilitate their dissemination, with adoption efforts accelerating markedly after 2022. In parallel, WCR coordinated a large multi-country trial conducted across 18 countries and 29 sites, comparing 32 coffee varieties—including 30 pure-line cultivars and two F_1_ hybrids—under standardized evaluation protocols. The two F_1_hybrids, (‘Centroamérica’ and ‘EC16 Mundo Maya’), showed the best performance in terms of yield and resistance to coffee leaf rust (Gautron, 2025).

At the same time, private-sector actors have also invested in hybrid breeding. For example, Nestlé has developed its own F_1_ hybrids with the objective of producing varieties well adapted to the needs and constraints of smallholder farmers (Herrera, 2025). The results obtained in 9 coffee regions reinforce the increasing role of F_1_ hybrids as a key element of coffee production systems. Starbucks has expressed similar ambitions, supporting the development of improved coffee varieties aimed at strengthening productivity and resilience (https://www.dailymotion.com/video/x96pee8).

Until recently, the possibility of developing F_1_ hybrids instead of pure-line cultivars had never been seriously considered in Brazil, the world’s largest producer of *Coffea arabica* and the main global provider of Arabica cultivars. Yet this paradigm is now beginning to shift. Importantly, recent quantitative genetic studies in Brazil have provided robust statistical evidence for the existence of exploitable heterosis in Arabica. Medeiros et al. (2021) demonstrated significant general and specific combining ability effects in structured crosses among introgressed lines, confirming the presence of stable heterotic patterns. More recently, Andrade et al. (2025) reported substantial heterotic potential together with both additive and non-additive genetic variance components across diverse Arabica germplasm, further reinforcing the view that hybrid vigor in this species is both measurable and predictable.

When F_1_ hybrids are first evaluated in experimental station trials and directly compared with their parental lines, heterosis relative to the best parent can sometimes exceed +200%. Such large values partly reflect the low vigor of many pure-line parents, which have accumulated deleterious alleles through long-term selfing and narrow genetic bottlenecks. However, once the most promising hybrids are propagated clonally or through seed multiplication and evaluated under farmers’ conditions, the realized yield advantage is typically more moderate. Across multiple environments, average productivity gains generally range from 30 to 40% compared with the best commercial pure-line cultivars. Even at this level, the magnitude of hybrid advantage remains agronomically substantial for a perennial crop.

**Figure 1:**
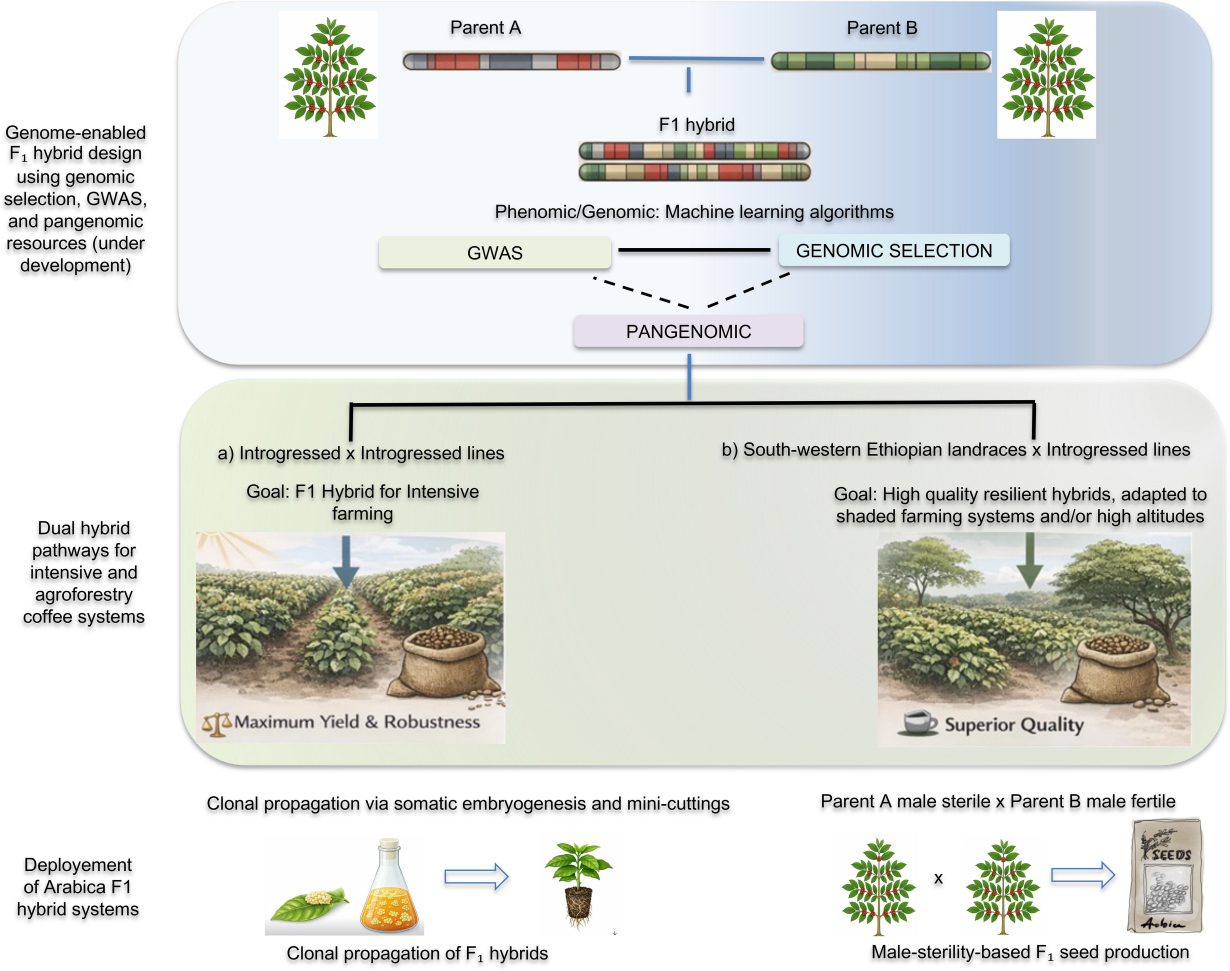
Genome-enabled design and deployment pathways for Arabica F₁ hybrids.This image summarises the conceptual framework linking the creation of hybrids, genomic-assisted breeding and large-scale deployment strategies in *Coffea arabica*. Two complementary breeding pathways are illustrated. The first combines introgressed Arabica lines carrying distinct *C. canephora*-derived chromosomal mosaics, generating highly productive hybrids adapted to intensive and full-sun systems. The second pathway combines introgressed lines with genetically differentiated southwestern Ethiopian landraces to produce productive and high quality resilient F_1_ hybrids adapted to shaded farming systems and/or high altitudes. These hybridisation schemes are progressively supported by genome-enabled breeding tools, including genome-wide association studies (GWAS), genomic selection, pangenomic resources and machine-learning–assisted phenomic approaches, which facilitate the identification of complementary parental combinations and the prediction of hybrid performance. The figure also illustrates the two main deployment strategies currently available for Arabica F₁ hybrids. Clonal propagation through somatic embryogenesis and mini-cuttings enables large-scale dissemination while preserving the complete F₁ genomic configuration. In parallel, male-sterility-based seed production systems provide a scalable alternative for hybrid seed deployment. However, seed-based diffusion remains constrained by the limited availability of validated male-sterile sources. To date, a single Ethiopian-derived male-sterile source has enabled commercial hybrid seed production for the ‘Starmaya’ cultivar (Georget et al., 2019), and more recently for the ‘Mariana’ cultivar (unpublished). Additional male-sterile candidates recently identified in Colombia (Suárez and Flórez Ramos, 2023) and Nicaragua (unpublished), together with single nucleotide polymorphism (SNP) markers co-segregating with male sterility (Mieulet et al., 2025), are expected to broaden hybrid seed production.

Together, these developments illustrate the progressive transition from experimental hybrid breeding toward genome-enabled hybrid deployment strategies adapted to contrasting coffee production systems. The main breeding pathways, genomic design tools, and dissemination platforms currently supporting Arabica F_1_ hybrid development are summarized in **Figure 1**.

Yet despite these substantial productivity gains, F_1_ hybrids still represent only a small fraction of the billions of coffee trees currently planted worldwide. Although they remain marginal in commercial landscapes, they have rapidly become focal points of research for coffee breeders, quantitative geneticists, and crop physiologists. The pace of recent advances in their development, evaluation, and dissemination suggests that Arabica cultivation may be approaching a major shift in breeding strategy. If this trajectory continues, the adoption of F_1_ hybrids could ultimately reshape Arabica improvement in a way comparable to the hybrid revolution that transformed maize agriculture during the twentieth century (Troyer, 2009).

This emerging transition toward hybrid breeding also raises a fundamental question. *Coffea arabica* is a predominantly selfing allopolyploid species that experienced a severe domestication bottleneck and therefore possesses one of the narrowest genetic bases among major crops, yet recent breeding programs have revealed strong and reproducible heterosis in F_1_ hybrids. To understand this apparent paradox, we first examine how functional diversity can arise despite limited allelic variation and discuss its implications for adaptive potential (Section 2). We then describe the existence of two complementary heterotic systems in *Coffea arabica* and explore how genomic tools can help structure heterotic groups (Sections 3, 4). Finally, we discuss the transition from experimental hybrids to scalable hybrid agriculture, propose a general framework for hybrid activation, and highlight key research gaps and future directions (Sections 5–7).

## 3 Functional diversity without allelic breadth: adaptive limits and regulatory potential

Allopolyploid crops often combine initial novelty from interspecific genome merger with subsequent bottlenecks that reduce nucleotide diversity. As a result, genomes can be structurally complex yet genetically uniform at the SNP level. In *C. arabica*, genome-wide diversity is exceptionally low (Anthony et al., 2001; Salojärvi et al., 2024; Scalabrin et al., 2020), yet phenotypes vary across environments, consistent with functional diversity emerging from homeolog retention, regulatory divergence, dosage interactions, and epigenetic modulation rather than from abundant allelic polymorphism.

### 3.1 Subgenome retention and regulatory differentiation

Following allopolyploidization, duplicated homeologs may diverge in expression, regulation, and epigenetic state. In Arabica, evidence for homeolog expression bias and environment-dependent non-additive transcription suggests regulatory plasticity that can modulate physiology without requiring extensive new alleles (Lashermes et al., 2014, 2016; Combes et al., 2012).

### 3.2 Dosage balance and transcriptional homeostasis

Dosage-sensitive pathways constrain but also stabilize gene expression networks, promoting transcriptional homeostasis (Bardil et al., 2011). Context-specific homologue expression bias evidenced during seed development or thermal regimes and in pathways such as caffeine biosynthesis (Combes et al., 2013 and 2023), illustrates how allopolyploidy has generated emergent functional diversity through regulatory specialization while maintaining overall genome stability. Such buffering can sustain performance across moderate environmental variation (Bertrand et al., 2015), yet it cannot fully substitute for allelic innovation when novel resistance or stress-adaptation loci are required.

### 3.3 Adaptive ceilings and the need for activation

**Figure 2:**
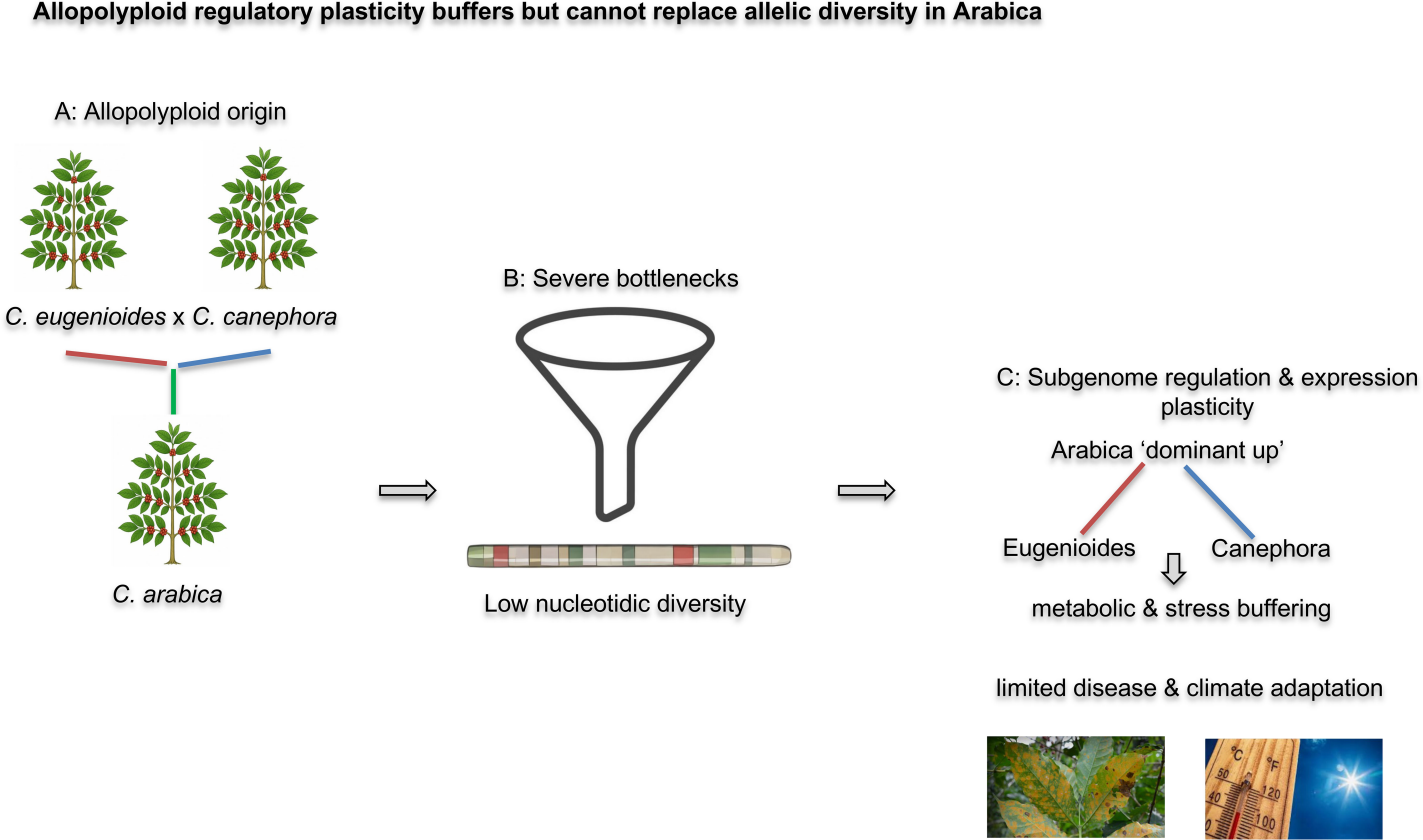
Diagram illustrating the evolutionary history and genetic consequences in Coffea arabica: Panel **(A)** depicts allopolyploid origin from C. eugenioides and C. canephora, panel **(B)** shows a genetic bottleneck causing reduced nucleotide diversity, and panel **(C)** explains regulatory plasticity from subgenome interactions buffering stress but resulting in limited disease and climate adaptation, supported by small photos of rust-diseased leaves and a sunlight thermometer.

The same processes that buffer phenotypes can impose adaptive ceilings in bottlenecked systems. Hybridization can shift the system from buffering to activation by perturbing and re-optimizing subgenome interactions or by combining complementary introgressed blocks (Scalabrin et al., 2024; Salojärvi et al., 2024) that expand effective functional diversity. These relationships between allelic bottlenecking, regulatory buffering, and adaptive limits are summarized in **Figure 2**.

These observations suggest that the same allopolyploid regulatory architecture that buffers phenotypic variation in pure Arabica lines may also provide a latent substrate for hybrid activation.

**Figure 3:**
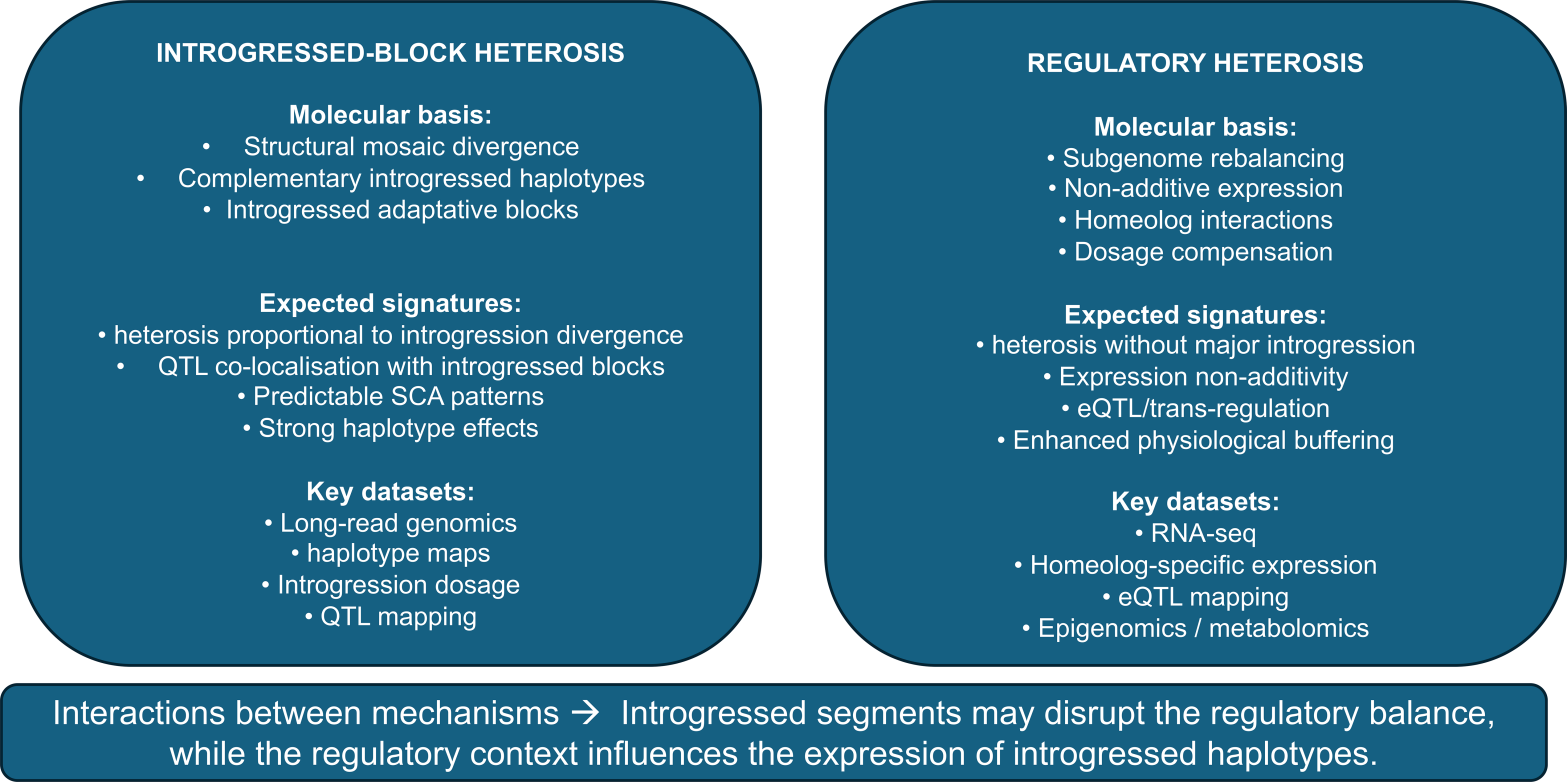
Contrasting Mechanisms of Heterosis in *Coffea arabica*. Diagram comparing introgressed-block heterosis and regulatory heterosis, detailing their molecular basis, expected signatures, and key datasets. Introgressed-block heterosis involves structural mosaic divergence and adaptive blocks, using long-read genomics, haplotype maps, and QTL mapping. Regulatory heterosis concerns subgenome rebalancing, dosage compensation, and non-additive expression, relying on RNA sequencing, eQTL mapping, and epigenomics. Both mechanisms interact, with a note stating introgressed segments may disrupt regulatory balance while regulatory context influences haplotype expression.

The key question then is how hybridization can convert this buffered, genetically narrow background into enhanced vigor, stability and adaptive capacity. We propose that crossing exploits this hidden potential through two complementary mechanisms: the recombination of divergent, introgressed chromosomal mosaics, and the rebalancing of regulatory interactions between ancestral subgenomes. The molecular basis and expected experimental signatures of these two mechanisms are summarized in **Figure 3**.

## 4 Two complementary heterotic systems in *Coffea arabica*

### 4.1 Introgressed-block heterosis

Breeding-mediated introgression from *C. canephora* has introduced large chromosomal segments into Arabica breeding materials, generating stable genomic mosaics within Arabica genetic backgrounds. Crosses between parents carrying complementary mosaics can produce substantial and often predictable heterosis, because the functional unit of divergence corresponds to extended chromosomal blocks containing multiple linked loci rather than isolated SNPs (Scalabrin et al., 2024). In particular, crosses between introgressed pure-line varieties and southwestern Ethiopian genotypes likely benefit from the complementation of adaptive traits carried by these divergent genetic backgrounds. This genetic complementarity can translate into superior agronomic performance under field conditions and supports the deployment of F_1_ hybrids as a promising strategy to enhance the resilience of coffee agroecosystems to climate change (Pappo et al., 2021; Kahsay et al., 2023). In bread wheat, breeding-mediated introgressions from wild relatives have generated mosaics of divergent haplotypes across the genome. Their impact on wheat improvement has been relatively modest. The major reason for this outcome is that many of genotypes have proven to be defective in terms of plant type, grain yield and/or grain quality, reflecting a combination of linkage drag and an inadequate level of genetic complementation (Hao et al., 2020). This limitation does not appear to apply to Arabica coffee, likely because of its close evolutionary relationship with *Coffea canephora*, which may facilitate functional compatibility and adaptive complementation between introgressed genomic regions.

### 4.2 Regulatory heterosis

Beyond introgressed-block heterosis, *Coffea arabica* also appears capable of expressing hybrid vigor in the absence of recent interspecific introgression, a phenomenon that can be interpreted as allopolyploid regulatory heterosis. Despite severe historical bottlenecks (Bawin et al., 2021; Salojärvi et al., 2024), pronounced population structure persists between Ethiopian and Yemeni Arabica gene pools (Anthony et al., 2001; Scalabrin et al., 2024; Medrano et al., 2025). These lineages have likely experienced contrasting ecological and agronomic selection regimes over several centuries (Montagnon et al., 2025).

Since at least the fourteenth century, Yemeni Arabica has probably been cultivated under full-sun, high-altitude conditions characterized by marked dry seasons (Montagnon et al., 2021). In contrast, southwestern Ethiopian Arabica consists largely of wild or semi-domesticated populations adapted to shaded, humid montane forest environments (Labouisse et al., 2008).

Such divergent ecological contexts are expected to have shaped distinct physiological and regulatory trait complexes, including differences in water-use efficiency, photoprotection capacity (Toniutti et al., 2019), carbon assimilation dynamics, and stress-tolerance mechanisms. Even in the absence of substantial nucleotide divergence, prolonged exposure to contrasting selection pressures may have led to differentiated regulatory architectures between these gene pools.

Comparable processes have been documented in other crops. In maize, long-term selection under contrasting agronomic conditions contributed to the emergence of distinct heterotic groups (Duvick, 2005), while in rice, divergent ecological adaptation among varietal groups helped structure the parental pools used in hybrid breeding (Huang et al., 2015). Highly vigorous F_1_ hybrids can be obtained by crossing introgressed Yemeni-derived lines with southwestern Ethiopian germplasm (Bertrand et al., 2005; Echeverria-Beirute et al., 2017). In such combinations, heterosis likely results from both the recombination of divergent introgressed haplotypes and the regulatory divergence accumulated between historically distinct Arabica gene pools. This illustrates how structural and regulatory mechanisms may jointly contribute to hybrid activation within a genetically constrained allopolyploid background.

We therefore propose that hybrids derived from these structured populations may express vigor through regulatory rebalancing between ancestral subgenomes. Non-additive gene expression, shifts in homeolog expression bias, and improved coordination of duplicated regulatory modules may enhance metabolic integration, resource allocation efficiency, and environmental buffering capacity. In this framework, heterosis arises not only from allelic complementation, but also from the transient optimization of regulatory networks embedded within the allopolyploid genome (Chen, 2010).

### 4.3 Boundaries and interactions

These two heterotic systems are not mutually exclusive. Introgressed genomic blocks may alter gene dosage relationships and perturb subgenome balance, while the surrounding regulatory context modulates the phenotypic expression of these segments. Structural mosaic complementarity and regulatory rebalancing therefore likely interact in a context-dependent manner rather than operating as independent mechanisms (**Figure 3**).

Similar interactions have been reported in other crops. In bread wheat, breeding-mediated introgressions from wild relatives have generated mosaics of divergent haplotypes across the genome (Hao et al., 2020). In tomato, large chromosomal segments introgressed from wild species have reshaped elite germplasm and contributed to agronomic diversification despite a narrow domestication bottleneck (Lin et al., 2014). Disentangling their relative contributions will require crossing designs that explicitly contrast introgression content and population structure across multiple environment (see **Table 1**).

**Table 1:** Crossing design to distinguish mechanisms.

Crossing design	Main comparison	Evidence supporting introgressed_bloc heterosis	Evidence supporting regulatory heterosis	Key analyses
Introgressed × introgressed	Parents carrying different *C. canephora* mosaics	Strong heterosis associated with complementary introgressed haplotypes	Possible if hybrids also show non-additive expression	Haplotype mapping, introgression dosage, QTL, GCA/SCA
Introgressed × non-introgressed	Structural mosaic × divergent Arabica background	Heterosis linked to presence/absence of introgressed blocks	Heterosis linked to population regulatory divergence	SNP/structural markers, RNA-seq, physiological traits
Non-introgressed Ethiopian × non-introgressed Yemeni lines	Divergent Arabica backgrounds without major introgression	Weak support unless hidden structural variants are detected	Strong support if heterosis occurs with non-additive expression	Transcriptomics, eQTL, homeolog expression bias
Reciprocal crosses	Same nuclear genome, different cytoplasmic direction	Limited effect expected unless cytoplasm interacts with introgressions	Maternal/cytoplasmic or dosage-related regulatory effects possible	Reciprocal hybrid performance, cytoplasmic haplotypes
Multi-environment trials of the same hybrid set	Stability across climates and management systems	Stable effects if linked to resistance/adaptive introgressions	Strong G×E buffering and homeostasis expected	Variance partitioning, G×E models, stability indices

Similar ambitions to capture or stabilize favorable heterotic configurations have been pursued in other allopolyploid crops, including cotton and oilseed rape. In oilseed rape, the pyramiding effect of heterotic QTL and the multiplicative effect of individual component traits could well explain substantial parts of yield heterosis (Ye et al., 2023). However, fixing heterotic effects remains complex because hybrid performance frequently depends on non-additive interactions, structural complementarity, dosage balance and environmental responsiveness. Arabica therefore provides a stringent perennial model in which these broader allopolyploid breeding challenges can be examined.

## 5 Genome-enabled structuring of heterotic groups

### 5.1 Mapping introgression mosaics with genome resources

Chromosome-scale assemblies and dense molecular markers now allow breeders to identify and map introgressed segments and to quantify mosaic complementarity among candidate parents. In coffee, high-quality reference genomes and population genomic resources are rapidly expanding and should improve the resolution of structural variation and haplotypes as curated community datasets become available (Scalabrin et al., 2020; Salojärvi et al., 2024). Pangenome-scale resources are being developed and should improve the resolution of structural variation and haplotypes once curated community datasets become available (preliminary results Boukhari et al., 2025).

### 5.2 GWAS and functional marker integration

GWAS approaches (Sant’Ana et al., 2018) and other genotype–phenotype association analyses can help identify introgressed regions and regulatory loci contributing to agronomic performance, thereby guiding the choice of parental combinations based on functional complementarity rather than phenotype alone. Polyploid-aware models remain essential to account for linkage disequilibrium and subgenome effects.

### 5.3 Genomic prediction for hybrid performance

Genomic prediction can forecast hybrid yield and stability from parental profiles (Carvalho et al., 2020; Mbebi et al., 2022) reducing reliance on exhaustive diallels. Models that incorporate additive and non-additive effects are especially relevant for capturing both block complementarity and regulatory heterosis.

In practice, GWAS-derived markers can be used to identify parental lines carrying complementary favorable alleles or introgressed haplotypes for key traits such as rust resistance, yield stability, bean quality and stress adaptation. Genomic prediction models can then estimate the expected performance of hybrids before field testing, particularly when the models include non-additive terms such as dominance, epistasis and specific combining ability. Together, these tools enable breeders to transition from empirical crossing to rational heterotic group design.

## 6 From experimental hybrids to scalable hybrid agriculture

Strong heterosis does not automatically result in an agronomic impact in crops that predominantly self-fertilize. Demonstrating consistent hybrid advantage requires rigorous, multi-location trials that are designed to quantify genotype-by-environment (G×E) interactions, as well as to assess yield stability under contrasting climatic and management conditions (Marie et al., 2020; Berny Mier y Teran et al., 2025). As hybrid performance can vary across environmental gradients, especially under stressful conditions, a robust evaluation across representative agroecological zones is crucial to distinguish transient vigor from stable agronomic superiority.

Large-scale deployment can only be considered once performance stability has been established. Effective hybrid dissemination requires reproductive control systems and multiplication platforms capable of preserving F_1_ genomic configurations at economically viable scales. In predominantly self-fertilizing perennial crops, this relies on either reliable clonal propagation systems or male-sterility-based hybrid seed production strategies that ensure genetic purity and operational feasibility.

### 6.1 Clonal propagation

Somatic embryogenesis and vegetative multiplication provide an efficient way to deploy elite F_1_ genotypes without the need for repeated controlled pollination. This preserves the complete combination of introgressed blocks and regulatory interactions that underlie hybrid performance. However, large-scale clonal deployment requires strict quality control procedures to minimize somaclonal variation and maintain genetic fidelity (Etienne et al., 2018).

### 6.2 Male sterility and hybrid seed systems

Male sterility offers an attractive route for scalable hybrid seed production, as it eliminates the need for emasculation and reduces self-fertilization (Georget et al., 2019; Suárez and Flórez Ramos, 2023). Marker-assisted tracking of sterility alleles could improve the efficiency and reliability of hybrid seed pipelines, and genome editing may eventually facilitate the targeted engineering of reproductive control systems (Breitler et al., 2018; Farinati et al., 2023).

However, current Arabica hybrid seed systems remain vulnerable as they depend on a very limited number of validated male-sterile sources. This narrow reproductive control base represents a major bottleneck for large-scale deployment and increases the fragility of hybrid breeding pipelines. Although genome editing has been shown to work in coffee, developing commercially viable male sterility systems remains a prospect. Such systems will require further technical validation, stable inheritance across genetic backgrounds, regulatory approval and societal acceptance before they can be realistically considered for commercial implementation.

More broadly, integrating structural genomic analyses, association mapping and genomic prediction should progressively enable the rational design and deployment of Arabica F_1_ hybrids adapted to contrasting production systems, including intensive cultivation and agroforestry-based coffee systems (**Figure 1**).

## 7 Toward a general framework for hybrid activation

In bottlenecked allopolyploids, heterosis can be viewed as hybrid activation: the deliberate reconfiguration of structural and regulatory complementarity embedded in duplicated genomes.

Activation is expected to operate within an optimal divergence window: too little divergence yields limited complementarity, whereas too much divergence risks incompatibility or dosage imbalance. Genome-enabled design aims to locate this window and to assemble parent pairs that maximize activation while maintaining stability across environments.

**Figure 4:**
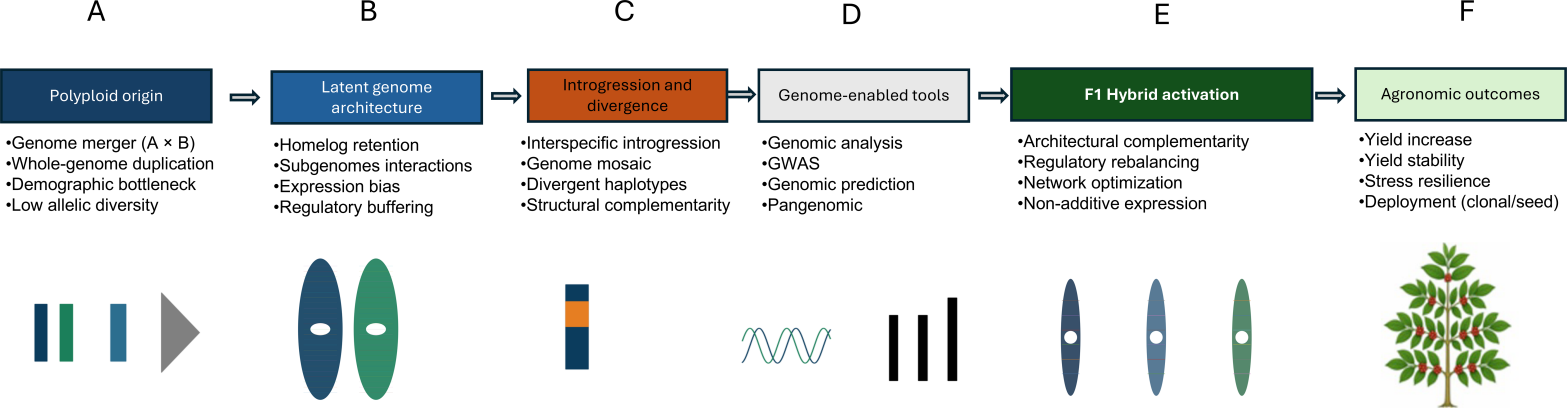
Hybrid activation framework in genetically constrained allopolyploid crops. Diagram outlining six sequential stages **(A–F)** in crop improvement: Polyploid origin, Latent genome architecture, Introgression and divergence, Genome-enabled tools, F_1_ hybrid activation, and Agronomic outcomes, with associated icons and explanatory text for each step.

Although developed from observations in *Coffea arabica*, the hybrid activation framework proposed here may extend more broadly to other allopolyploid crops characterized by restricted allelic diversity, subgenome regulatory interactions, and episodic introgression. **Figure 4** summarizes this generalized conceptual model linking allelic bottlenecks, regulatory buffering, introgressed genomic mosaics, and hybrid activation across allopolyploid breeding systems.

Translating this conceptual framework into a predictive and operational breeding strategy now requires experimental validation across genomic, physiological, and agronomic scales. Several major research questions therefore remain to be addressed.

## 8 Research gaps and future directions

In order to transform the proposed hybrid activation framework from a conceptual model into a predictive breeding strategy, several key questions must first be addressed.

### 8.1 Quantifying the relative contributions of introgressed blocks and regulatory heterosis

Firstly, the relative contributions of introgressed blocks and regulatory heterosis must be quantified. Disentangling these contributions will require integrative experimental designs combining quantitative genetics, structural genomics, transcriptomics, and multi-environment phenotyping (**Table 1**). At the quantitative genetic level, structured factorial or diallel populations should allow the partitioning of variance components for additive effects, dominance effects, epistatic effects, general combining ability (GCA), specific combining ability (SCA), and genotype-by-environment interaction. Strong associations between hybrid performance and parental introgression divergence, haplotype complementarity, or introgression content covariates would support the idea that introgressed-block heterosis makes a significant contribution. Conversely, persistent non-additive effects after accounting for introgressed genomic content would suggest that regulatory mechanisms make a significant contribution.

### 8.2 Resolving the structure and functional significance of introgressed mosaics

The stability, boundaries, dosage and functional content of introgressed chromosomal segments must be determined using long-read sequencing and haplotype-resolved assemblies, as well as future coffee pangenome resources. These methods should clarify whether the functional units underlying heterosis correspond primarily to extended structural haplotypes or isolated SNP effects. Improved structural resolution will also facilitate the identification of recombination breakpoints, linkage drag, and favorable introgression combinations that contribute to hybrid performance.

### 8.3 Modelling genotype-by-environment interactions and hybrid stability

Multi-environment phenotyping is essential for determining the extent to which regulatory heterosis contributes to environmental buffering and yield stability under stressful conditions. By contrast, introgressed-block heterosis may contribute more directly to resistance or adaptive trait complementation. Integrating physiological, agronomic, and environmental datasets should improve predictions of hybrid stability across contrasting agroecological conditions and help identify the environmental contexts in which hybrid activation is maximized.

### 8.4 Integrating genomic, regulatory and phenotypic datasets

Combining genomic, regulatory and phenotypic datasets within the same hybrid populations should allow us to estimate whether hybrid performance primarily reflects chromosomal mosaic complementarity, regulatory rebalancing between ancestral subgenomes or the dynamic interaction between these two mechanisms. Combining haplotype-resolved genomics, RNA-seq, homeolog-specific expression analyses, eQTL mapping and physiological profiling may provide a unified framework for understanding how structural and regulatory diversity jointly shape hybrid vigor in allopolyploid crops.

An important challenge will be to establish practical genomic distance metrics and optimal divergence windows for hybrid activation within breeding populations. Excessively narrow divergence may limit complementarity, whereas excessive divergence could generate incompatibilities, dosage imbalances, or instability. Genome-enabled breeding approaches may therefore help identify intermediate levels of structural and regulatory divergence that maximize hybrid performance while maintaining agronomic stability.

### 8.5 Integrating reproductive innovation with breeding deployment

Future breeding strategies must integrate reproductive innovations, such as male sterility systems and clonal propagation platforms, alongside genomic design and socio-economic feasibility. While genome editing is now possible in coffee, the development of commercially deployable male sterility systems is still in the future. These systems will require further technical validation, regulatory approval, and societal acceptance before they can be implemented on a large scale. Meanwhile, current hybrid seed systems remain dependent on a very limited number of male-sterile sources, which highlights the need to diversify reproductive control resources in order to ensure the long-term robustness of hybrid breeding pipelines.

Validation beyond coffee will be critical to assess the broader relevance of the hybrid activation framework. Comparative studies in other allopolyploid crops, such as cotton (He et al., 2020; Feng et al., 2021), oilseed rape, wheat, and sugarcane (Mahadevaiah et al., 2021; Healey et al., 2024), could clarify whether heterosis predominantly emerges from introgressed genomic mosaics, subgenome regulatory rebalancing, or dynamic interactions between these mechanisms. They may also reveal whether comparable breeding principles generalize across diverse polyploid crop systems.

## 9 Concluding perspective

Allopolyploid crops are often portrayed as genetically exhausted because of bottlenecks and selfing, yet their duplicated genome architecture preserves structural and regulatory complexity that can remain partially dormant under pure-line regimes. In *Coffea arabica*, structured hybridization activates this latent potential through complementary introgression mosaics and subgenome regulatory rebalancing. Genome-enabled breeding and reproductive technologies now make it realistic to move from empirical discovery to predictable hybrid design and deployment.
